# Cardiopulmonary resuscitation of a cardiac arrest patient with left ventricular assist device in an out-of-hospital setting: A case report

**DOI:** 10.1097/MD.0000000000018658

**Published:** 2020-01-10

**Authors:** Yoshiaki Iwashita, Asami Ito, Ken Sasaki, Kei Suzuki, Masaki Fujioka, Kazuo Maruyama, Hiroshi Imai

**Affiliations:** a Emergency and Critical Care Center, Mie University Hospital; b Department of Anesthesiology and Critical Care Medicine, School of Medicine, Mie University, 2-174 Edobashi, Tsu, Mie 514-8507, Japan.

**Keywords:** cardiopulmonary arrest, CPR, ECMO, LVAD

## Abstract

**Rationale:**

Despite increasing number of left ventricular assist device (LVAD) implantation, standardized cardiopulmonary resuscitation (CPR) protocol for patients with LVAD, especially in out-of-hospital settings are not well known.

**Patient concerns:**

A 41-year-old LVAD implanted man became cardiac arrest in an out-of-hospital setting. Bystander CPR was started and the patient was brought to our hospital without noticing LVAD. Upon arrival, the medical staff noted the LVAD and that the battery of the LVAD was exhausted.

**Diagnosis:**

Cardiac arrest on LVAD.

**Interventions:**

It took 50 minutes to change the battery, then the patient has become ventricular fibrillation; hence, we introduced extracorporeal membranous oxygenation and defibrillated the patient. After the sinus rhythm was restored, the LVAD started working uneventfully.

**Outcomes:**

The patient became brain dead.

**Lessons:**

There are several difficulties in treating these patients. First, hemodynamic collapse is difficult to diagnose. Second, chest compression for LVAD implanted patients remains controversial. Third, education to first responders who are not familiar with LVAD are not enough. Appropriate education for those issues is needed.

## 1 Introduction

The number of patients with left ventricular assist device (LVAD) is increasing and has reached 2500 implants per year in 2016 in the United States.^[1]^ Despite the increasing number of LVAD implantations, standard emergency protocol for the management of patients with LVAD is not well established. Many of the current LVADs are nonpulsatile; thus, it is difficult to recognize hemodynamic deterioration. Upon onset of hemodynamic deterioration, chest compression for those patients is generally contraindicated.^[2]^ It is even difficult for emergency medical services (EMS) and physicians who are working in hospitals that do not perform LVAD implantation. In Japan, the number of heart transplantation in 2013 was only 37 cases and the national insurance policy does not cover LVAD implantation as a destination therapy.^[3]^ Therefore, LVAD implantation surgery is performed in limited regions. We encountered a rare case of a patient who collapsed due to LVAD battery exhaustion in an out-of-hospital setting in a place where LVAD implantation is not common.

## 2 Case presentation

A 41-year-old man, who had LVAD (HeartMateII©) implantation for severe dilated cardiomyopathy 2 years prior at a hospital 200 km away from his permanent residence, collapsed at a pachinko (a Japanese gambling parlor), where a loud music was playing. The patient was unaccompanied by a caregiver. Bystander CPR was started soon on the scene, and the patient was brought to our hospital, with CPR performed by the EMS about 40 minutes after collapse. Although the EMS noticed an alarm sound, they were not able identify that it was coming from the LVAD, as LVAD implantation is not common in our region.

Upon arrival at our hospital, the initial rhythm of the patient was pulseless electrical activity. We continued performing CPR, during which we noticed that the patient had LVAD and that the alarm sound was coming from it. We also found LVAD batteries in the patient's bag; thus, we suspected that the sound signified battery exhaustion. It is probable that the low battery alarm cannot be heard at a pachinko, and the patient received a cardiac arrest.

**Figure 1:**
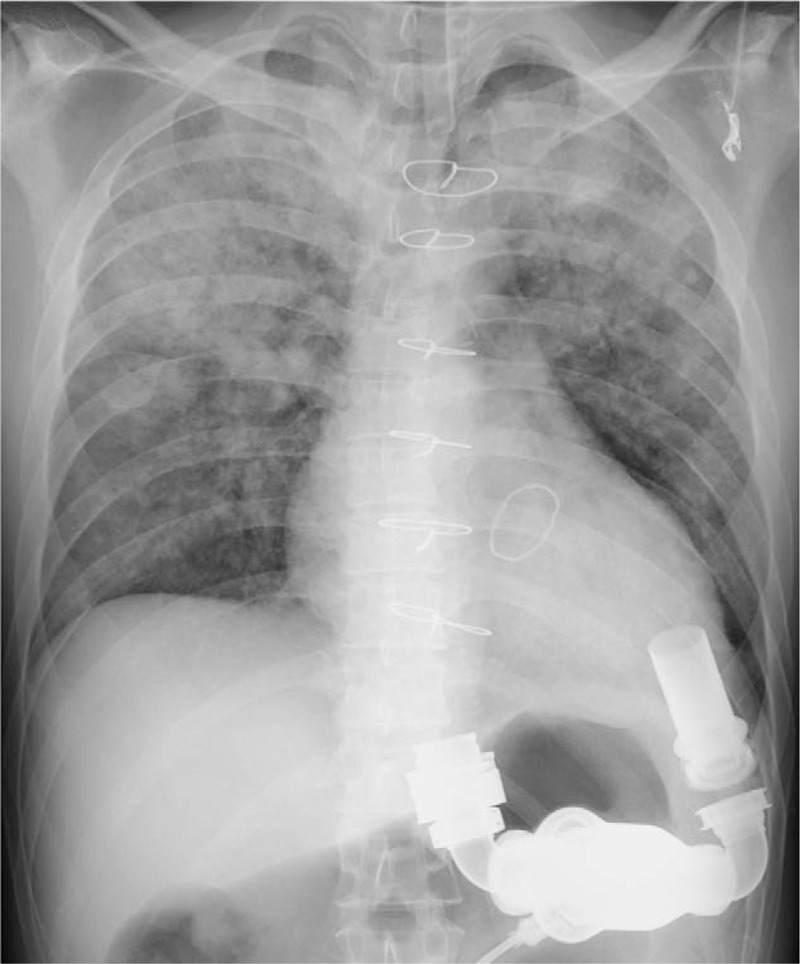
Chest X-ray showing diffuse bilateral infiltrates. There was no dislodgement of the LVAD.

**Figure 2:**
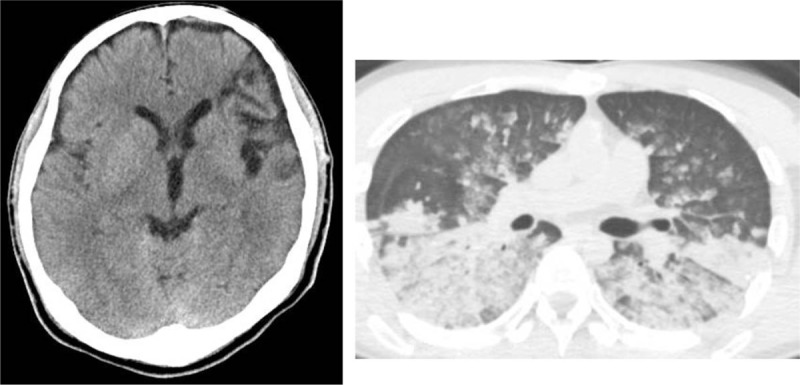
Head computed tomography (CT) showing no signs of hypoxic brain injury. Chest CT showed bilateral diffuse infiltrate suggesting cardiogenic pulmonary edema.

We changed the battery, at approximately 50 minutes after collapse, and the alarm sound stopped. Chest X-ray showed bilateral diffuse infiltrates (Fig. 1). Arterial blood gas analysis showed the following results: pH 6.8, pCO_2_ 93 mmHg, lactate 18 mmol/L, potassium 5.4 mEq/L. Ultrasound cardiography showed little flow through the LVAD. We stopped CPR and performed computed tomography (CT). Head CT showed no signs of hypoxic brain injury, and chest CT showed bilateral dependent lung area injury (Fig. 2). After the CT scan, the patient had ventricular fibrillation (VF), and ultrasound cardiography (UCG) showed no activity in the right ventricle. We tried to defibrillate the patient, but the patient did not recover to sinus rhythm. Therefore, we decided to introduce venoarterial extracorporeal membranous oxygenation (VA-ECMO) about 2 hours from collapse. A 20-Fr venous catheter was inserted in the right femoral vein and a 16-Fr arterial catheter was inserted in the left femoral artery. After initiating ECMO, the serum potassium level increased to 7.0 mEq/L, which was possibly due to successful reperfusion. We treated the hyperkalemia and subsequently defibrillated the patient. Then the sinus rhythm was restored, and the LVAD started working uneventfully. We transferred the patient to a hospital where LVAD implantation was performed. However, when the ECMO was removed, the patient became brain dead.

## 3 Discussion

In our prefecture, only a few patients have LVAD, and neither EMS personnel nor our hospital staffs are familiar with the device; thus, it took a long time before ECMO was introduced in our patient. There are several problems in treating LVAD patients who has become cardiac arrest: recognition of cardiac arrest, feasibility of chest compression, and education of first responders who are not familiar with LVAD.

Recognition of cardiac arrest of patients with LVAD is difficult because of the constant flow of the LVAD, which resulted in non-pulsatile patient. According to the AHA scientific statement, once LVAD is recognized, assess the adequate perfusion by skin color and capirally refil, then listen for the alarm and LVAD hum sound are recommended.^[4]^ Some reports indicate that collapsed patients with LVAD should be assessed by Doppler echo to determine pulsation.^[5,6]^ However, the recognition of LVAD itself is difficult for regions where there are no LVAD implantable institutions. The use of Doppler might be possible in hospital settings; however, it is not feasible in out-of-hospital settings. In our prefecture, EMS are not trained to use Doppler because of the low LVAD population. Neither are general bystanders capable of using Doppler echocardiography. Another reason why hemodynamic collapse is difficult to establish is the difficulty of understanding the meaning of the alarm. Patients are advised to be accompanied by a caregiver to interpret the alarm; however, our patient went to the pachinko alone, and thus, the LVAD was not recognized by the first responders. Safety systems, such as visual display or voice message, are needed for easy understanding during first encounters.

The use of chest compression on patients with LVAD remains controversial. It is generally contraindicated because of the risk of LVAD dislodgement or regurgitation (from the aorta to the left ventricle) may occur.^[2]^ In one case report, the percutaneously implanted aortic valve was destroyed due to prolonged CPR.^[7]^ Some other reports indicated the safety of chest compression.^[8,9]^According to the AHA scientific statement, if LVAD is non-functioning or LVAD functioning with mean arterial pressure < 50mmHg or EtCO2 < 20 mmHg, chest compression are recommended, though safety and efficacy of chest compression is not established.^[4]^ A limited report indicated the potential benefit of abdomen-only CPR as an alternative to conventional CPR.^[10]^ Our case showed normal operation after chest compression; thus, our experience further supports the safety of chest compression to LVAD implanted patients. However, even if chest compression may be safe, its effectiveness remains controversial. Our patient became brain dead. The reasons that may be thought of are; prolonged resuscitation time, and inadequate cerebral flow due to the recirculation in LVAD circuit.

Education of first responders as well as physicians^[11]^ working in hospitals that do not perform LVAD implantation regarding the LVAD system should be established. Since the number of LVAD implantations is increasing, physicians should learn emergency management for such patients. EMS personnel should also be familiarized with the LVAD because, as first responders, they may encounter patients with LVAD, whom they might need to transport to hospitals that perform LVAD implantation.^[12]^

## 4 Conclusion

We experienced a case of out-of-hospital cardiac arrest in a patient with LVAD. Establishment of a standard management protocol and education of first responders, including physicians in hospitals that do not perform LVAD implantation, are needed.

## Acknowledgments

We would like to thank Editage (www.editage.jp) for English language editing.

## Author contributions

**Conceptualization:** Yoshiaki Iwashita.

**Supervision:** Kazuo Maruyama, Hiroshi Imai.

**Writing – original draft:** Yoshiaki Iwashita.

**Writing – review & editing:** Asami Ito, ken Sasaki, Kei Suzuki, Masaki Fujioka.

Yoshiaki Iwashita orcid: 0000-0002-7054-8448.
